# Wildlife translocation: the conservation implications of pathogen exposure and genetic heterozygosity

**DOI:** 10.1186/1472-6785-11-5

**Published:** 2011-02-01

**Authors:** Walter M Boyce, Mara E Weisenberger, M Cecilia T Penedo, Christine K Johnson

**Affiliations:** 1 Wildlife Health Center, University of California, One Shields Avenue, Davis, California 95616, USA; 2 U.S. Fish and Wildlife Service, San Andres National Wildlife Refuge, 5686 Santa Gertrudis Drive, Las Cruces, New Mexico 88012, USA; 3 Veterinary Genetics Laboratory, University of California, One Shields Avenue, Davis, California 95616, USA

## Abstract

**Background:**

A key challenge for conservation biologists is to determine the most appropriate demographic and genetic management strategies for wildlife populations threatened by disease. We explored this topic by examining whether genetic background and previous pathogen exposure influenced survival of translocated animals when captive-bred and free-ranging bighorn sheep (*Ovis canadensis*) were used to re-establish a population that had been extirpated in the San Andres Mountains in New Mexico, USA.

**Results:**

Although the free-ranging source population had significantly higher multi-locus heterozygosity at 30 microsatellite loci than the captive bred animals, neither source population nor genetic background significantly influenced survival or cause of death. The presence of antibodies to a respiratory virus known to cause pneumonia was associated with increased survival, but there was no correlation between genetic heterozygosity and the presence of antibodies to this virus.

**Conclusions:**

Although genetic theory predicts otherwise, increased heterozygosity was not associated with increased fitness (survival) among translocated animals. While heterosis or genetic rescue effects may occur in F1 and later generations as the two source populations interbreed, we conclude that previous pathogen exposure was a more important marker than genetic heterozygosity for predicting survival of translocated animals. Every wildlife translocation is an experiment, and whenever possible, translocations should be designed and evaluated to test hypotheses that will further improve our understanding of how pathogen exposure and genetic variability influence fitness.

## Background

Innate and adaptive immune responses evolved in vertebrates as a first and secondary line of defense, respectively, against a diverse and changing array of pathogenic organisms. The effectiveness of these immunologic responses, and hence the fitness of individuals, populations, and species, is driven by pathogen exposure history and the immunogenetic repertoire of major histocompatibility complex (MHC) genes and non-MHC genes [1,2]. Novel, highly virulent pathogens can overwhelm host immune responses not primed to their exposure, and such pathogens can be a strong selective force, reducing the distribution and abundance of a species over short timeframes (1-2 generations) through effects on survival and reproductive success [2]. Over multiple generations, a history of ongoing pathogen exposure theoretically should select for more resistant immunogenotypes that limit fitness impacts by responding effectively upon initial exposure (innate immunity) or re-exposure (adaptive immunity).

Bighorn sheep (*Ovis canadensis*) are a useful model for examining this interplay between disease, demography, and genetics. They are a polygynous, highly philopatric species found in small, fragmented populations in the mountainous regions of western North America [3]. They are highly susceptible to infectious disease, and outbreaks of disease regularly cause high morbidity and mortality [3,4]. The history of population die-offs dates back to European settlement of the western United States >200 yrs ago [3], indicating that novel pathogens were likely introduced by contact with domestic sheep (*Ovis aries*). Pneumonia epizootics appear to be driven by density-dependence, serving to constrain population size [5], and presumably selecting for the most fit genotypes. However, small populations of bighorn sheep also are prone to inbreeding and genetic drift, making it difficult to understand the relative importance of pathogen-mediated selection, drift, and inbreeding on genetic variability and fitness.

We approached this problem by testing whether genetic background and previous pathogen exposure influenced survival when animals from two different founder populations were simultaneously translocated into the San Andres Mountains (SAM) in New Mexico, USA. The SAM once supported the largest population of native bighorn sheep in the state. However, by the late 1990's, a combination of disease, mountain lion (*Puma concolor*) predation, and drought had reduced this population to the point of extinction, and translocation from captive and/or free-ranging herds was necessary to reestablish a self-sustaining population in the SAM. The two founder populations chosen for reintroduction were a genetically diverse free-ranging herd in the Kofa National Wildlife Refuge (KNWR), Arizona, and a less diverse captive herd in the Red Rock Wildlife Area (RRWA) that was originally derived from the native SAM population. In November 2002, 51 bighorn sheep were translocated into the SAM from the KNWR (n = 20) and the RRWA (n = 31), and 30 more bighorn were translocated from KNWR in November 2005. We examined genetic variation at 33 microsatellite loci to compare genetic variability, and we conducted population health analyses at the time of capture to assess infectious disease exposure. Radiocollared sheep released into the SAM were then monitored through February 2007 to determine survivorship and cause specific mortality.

## Methods

### Study animals

The SAM mountain range represents the largest amount of high quality bighorn sheep habitat in New Mexico, and the native SAM bighorn sheep population exceeded 200 animals prior to a psoroptic scabies epizootic that began in the late 1970s. The population remained around 30 animals through the early 1990's and then declined to a single ewe that was temporarily brought into captivity in 1999 for less than two weeks. Nine animals sampled just prior to extirpation of the population had low mean heterozygosity at MHC (0.075) and microsatellite (0.359) loci [6]. Immediately prior to the reintroduction effort that began in November 2002, the SAM population consisted of 4 rams that had been translocated from the RRWA as part of a sentinel disease study, and the single native ewe that was released along with the sentinel rams [7]. A total of 81 bighorn sheep were captured, sampled, and translocated to the SAM in 2002 and 2005 from RRWA and KNWR. In 2007, nine offspring from these animals were captured and sampled for genetics and disease surveillance in the SAM. The survival analysis presented in this paper focuses only on animals translocated to the SAM, and data from these 9 offspring born in the SAM are presented for descriptive purposes only.

The RRWA is a 500-hectare enclosure for bighorn sheep located in central New Mexico. The RRWA population was initiated in 1972 with founders from the SAM, and has served as the source for >260 bighorn sheep translocated within New Mexico since 1979 [8]. This population has been managed as a closed herd (no immigrants), and has low genetic diversity (0.36) relative to other free-ranging populations in the desert southwest (0.44-0.63) [9]. Genetic and disease samples for this study were collected when bighorn sheep were captured for translocation to the SAM in 2002 (n = 31). The size of the RRWA population was <100 animals at the time of capture and translocation.

The KNWR is located in southwestern Arizona and contains a free-ranging population of bighorn sheep that has significantly higher heterozygosity (0.60) than the RRWA captive population [9]. Like the RRWA population, the KNWR herd has been the source for transplants of hundreds of animals within Arizona and to other western states. Genetic and disease samples for this study were collected at capture from animals translocated to the SAM in 2002 (n = 21) and 2005 (n = 30). The population at KNWR has averaged about 700-800 animals since 1981, but dropped to <400 animals by 2006. Since 1979, >560 bighorn sheep have been translocated from KNWR.

### Genetic analysis

Genomic DNA was extracted from blood and genetic variability was assessed by examining 33 microsatellite loci (OarFCB128, MAF209, OarFCB304, MAF33, MAF48, MAF65, OarFCB11, MAF36, OarFCB266, ETH152 (= D5S2), DRB3 (MHC-linked loci), BMC1009, CELJP23, BM203, OARCP026, TGLA94, FCB193, IRBP, CELB9, BM6506, CELJP15, BM4107, CSRD247, HSC, INRA023, INRA063, INRA105, MAF214, McM527, OarAE129, OarCP49, OarFCB20, SPS113). Many of these loci have been examined in previous publications [6,9,10], and we have used them to examine heterozygosity in different populations of bighorn sheep across their range in North America (unpublished). Briefly, using fluorescently labeled microsatellite primers, microsatellites present in genomic DNA were amplified by PCR (polymerase chain reaction). PCR products were separated by capillary electrophoresis using an Applied Biosystems 3730 DNA Analyzer (Applied Biosystems, Foster City, CA), with a fluorescent-labeled base pair size standard in each lane. Image analysis and fragment size determination were carried out using STRand software [11]. Deviations from Hardy-Weinberg equilibrium (HWE) and allele frequencies were examined with GenePop [12].

Multi-locus heterozygosity (MLH) values were calculated for each animal as the preferred metric of genetic variability. Chapman et al. [13] showed that different genetic metrics (MLH, SH, IR) are highly correlated and non-independent, and they advocated the use of the simplest metric, MLH, in future studies of heterozygote-fitness correlations (HFC). For our multivariate analyses, MLH values were categorized into two categories using a cutoff > 0.5 to indicate a generally low or high level of genetic diversity.

### Assessment of Health and Disease Exposure Status

Antibodies are a marker of previous exposure and priming of the adaptive immune response to specific pathogens. Sera from blood collected at the time of capture were tested for the presence of antibodies to three viruses known to cause respiratory disease and pneumonia in bighorn sheep: bovine respiratory syncytial virus (BRSV), bluetongue virus (BTV), and parainfluenza-3 virus (PI3). Assays were performed at the California Animal Health and Food Safety laboratory in Davis, California, and results were classified as positive or negative. Results for BTV were reported as positive or negative; titers ≥ 1:20 were considered positive for BRSV, and titers > 1:16 were considered positive for PI3.

### Post-release Monitoring

A VHF radiocollar (Telonics Inc., Lotek Wireless Inc., Advanced Telemetry Systems, Inc., and Telemetry Solutions) with mortality sensor was placed on animals to be translocated at the time of capture to facilitate monitoring after their release in the SAM. Animals were transported to the San Andres National Wildlife Refuge in the SAM and released within 48 hrs of capture. Resight of individual animals by radio-signal was conducted by state and federal wildlife biologists through February 2007 to detect mortalities and determine specific causes of death. Mortalities were investigated immediately and a field investigation and necropsy typically performed within 72 hrs of death. Mortalities were classified as lion predation based on criteria of Hayes et al. [14], or as pneumonia based on gross lesions present in the lungs. All mortalities that could not be classified as predation or pneumonia based on field examination were categorized as unknown, but "other" than predation and pneumonia. The number of days each animal survived was calculated as the difference between the date of release and the date of death, or the date sheep were last observed. For individuals lost to follow up, the end date was the date individuals were last observed, and for sheep that were confirmed alive at the end of the study period, this date was February 1, 2007.

### Statistical Analysis

We used independent univariate and multivariate approaches to explore correlation within our dataset, and to identify factors most influencing pathogen exposure, number of days until death (or end of study period if survived) and death due to suspected pneumonia. Independent variables evaluated for their impact on these three outcomes included sex, age-class (< 3 yr, 3 to < 6 yr, ≥ 6 yr), source population (RRWA vs KNWR), year released (2002, 2005), exposure status to each pathogen (BRSV, BTV, PI3) at time of release, and genetic variability (MLH). Significant associations with outcomes variables, confounding, and effect modification were evaluated by stratified univariate analyses using exact categorical tests and the student's t test. The relationship between MLH (as a continuous variable) and age and sex class were evaluated by the two-way ANOVA in order to adjust for source population. Independent categorical variables were also examined for their relationship to loss to follow-up by the two-sided Fisher exact test to determine whether variables were related to censorship, and therefore could not be included in the survival analyses.

Binary logistic regression was used to evaluate whether independent variables were related to death due to pneumonia, which was the most common cause of death detected during the study period. Only sheep that died of suspected pneumonia or were confirmed alive at the end of the study were included in this analysis (n = 50). Sheep that died from predation or unknown causes were excluded from this analysis. Variables were selected by backward stepwise elimination (likelihood ratio test P < 0.1) and confidence intervals for the logistic model evaluating risk factors for pneumonia were calculated using conditional exact inference due to low sample size. Hosmer-Lemeshow goodness of fit test was used to estimate overall fit of the final logistic model.

For the survival analyses, median survival time was calculated for all risk factors of interest measured at time of translocation. The Kaplan-Meier product-limit estimator was used to estimate the survivor function, and the log-rank test [15] was used to test the equality of survivor function for each independent variable with groups considered significantly different if P < 0.1. Variables significantly associated with survival in these univariate analyses were evaluated for their relationship to failure rate (days until death) using the semi-parametric Cox proportional hazards regression. Sex was significantly associated with censorship so this variable was excluded from survival analyses. Variables were selected for the Cox proportional hazards model by manual backward elimination using a selection criterion of P ≤ 0.1 for terms to stay in the model, and all categories of any significant variable were retained in the model. The Breslow approximation method was used to handle tied failure times [16].The proportional hazards assumption based on Schoenfeld residuals was evaluated to determine if the relative risk for each variable of interest was the same in time for the duration of the study. All statistical analyses were performed using STATA SE 11.1 software (STATACorp, 4905 Lakeway Drive, College Station, Texas 77845 USA).

## Results

### Characteristics of Source Populations

Bighorn sheep translocated from RRWA were predominantly male (18/20), while 72% (34/47) of the sheep translocated from KNWR were female (Fisher exact P < 0.001). Age class was not significantly associated with source population (Fisher exact test P = 0.068), but twice as many sheep in the 3-6 yr old range were captured in KNWR (23/36) compared to RRWA (11/31). All sheep relocated to SAM in 2005 were from KNWR, but these newly introduced sheep were demographically similar and did not differ with regard to pathogen exposure or genetic diversity from sheep introduced from KNWR in 2002.

Serologic evidence of previous pathogen exposure varied substantially by source population for all pathogens, except PI3 (Table 1). Sheep from KNWR were more likely to be exposed to BRSV (14/36) compared to sheep from RRWA (4/31, Fisher exact P = 0.026). None of the sheep captured in KNWR had evidence of exposure to BTV, while 12/31 sheep from RRWA were seropositive to BTV (Fisher exact P < 0.001). Results from the univariate analysis stratified by source population indicated that age was a significant confounder related to BTV exposure for sheep from RRWA. In fact, older sheep (≥ 6 years) captured at RRWA were 12 times more likely to be exposed to BTV than younger age classes (two sided Fisher exact test, P = 0.012). Significant associations with age class were not detected for the other pathogens after adjusting for the effect of source population.

**Table 1:** Multi-locus heterozygosity (MLH) and prevalence of antibodies to pathogens among bighorn sheep from the Kofa National Wildlife Refuge (KNWR) in Arizona, the Red Rock Wildlife Area (RRWA) in New Mexico, and the San Andres Mountains (SAM) in New Mexico, USA.

	RRWA ^a^ 2002 n = 30	KNWR ^a^ 2002 n = 20	KNWR ^a^ 2005 n = 31	SAM ^a^ 2007 n = 9
**Pathogen Exposure ^b^**				
BTV	38.7%	0%	0%	11%
PI3	74.2%	33.3%	34.0%	22%
BRSV	19.4%	42.9%	34.0%	11%
**Genetic Diversity ^c^**				
MLH	0.39	0.57	0.60	0.59

**^a ^**Bighorn sheep from RRWA and KNWR were sampled at the time of their translocation into the SAM (2002, 2005), and offspring from these animals were sampled in 2007 in the SAM.

**^b ^**BTV = bluetongue virus, PI3 = parainfluenza-3 virus, BRSV = bovine respiratory syncitial virus

**^c ^**Based on analysis of 30 microsatellite loci

Three of the 33 microsatellite loci tested were monomorphic and were excluded from further analyses (BM4107, INRA063, TGLA94). Complete genotypes were determined for all animals at each of the remaining 30 loci with the exception of a single animal at a single locus. Allele frequencies did not significantly differ from HWE expectations across loci, and null alleles were not detected. The genetic diversity parameter, MLH (Table 1), was significantly higher in sheep captured in KNWR (mean = 0.579, 95% CI 0.555 - 0.603) compared to sheep captured in RRWA (mean = 0.394, 95% CI = 0.340-0.449). Mean MLH did not differ by sex or age class once comparisons were adjusted by source population.

### Specific Causes of Mortality

Cause of death could be determined by field post-mortem examinations for 25 sheep. Pneumonia was suspected as a cause of mortality in 17 sheep, and mountain lion predation was identified as the cause of death in 8 sheep. Specific cause of death could not be determined for 9 sheep, but predation and pneumonia were excluded as their cause of death. Deaths due to lion predation occurred in 2002, 2003, and 2006, while deaths due to pneumonia occurred every year (2002-2007). Most deaths due to pneumonia (65%; 11/17) occurred in the fall from September thru November, and seven of these occurred in a cluster in the fall of 2006. Pneumonia impacted all age classes with 27% (4/15) of 1-3 year old sheep, 35% (9/26) of 3-6 year old sheep and 44% (4/9) of > 6 year old sheep dying of pneumonia. Mean MLH was not significantly different among the 33 sheep that survived (mean = 0.50, 95% CI 0.45 - 0.55) compared to the 17 sheep that died of pneumonia (mean = 0.44, 95% CI 0.37 - 0.51), but this analyses lacked power due to low sample size (two-sided t test P = 0.161, power = 0.31). Similarly, risk factors were not significantly associated with death due to pneumonia in the multivariate analyses using exact conditional logistic regression.

### Risk Factors Associated with Survival

Year of release was not associated with overall survival in univariate or multivariate analyses and survival time did not differ significantly by source population. However, age class at time of release was marginally associated with survivorship (log rank test P = 0.079) in the univariate analyses. More than half of the sheep released at 1 to < 3 years old survived the entire study period (1,509 days), while median survival time was 1,234 days for sheep 3 years to < 6 years old and only 475 days for sheep released when 6 years or older. Genetic diversity and pathogen exposure were not related to survival time, even after stratifying by source population.

Risk factors found to be significantly associated with time to death in the Cox proportional hazards multivariate framework were the oldest age class and previous exposure to BRSV (Table 2). Based on the hazards ratios of the Cox proportional hazards model, we found that sheep with serological evidence of previous exposure to BRSV had approximately one-third the risk of death compared to sheep that had not been exposed to BRSV prior to release. Age was a significant confounding factor in the survival analyses, and as expected, older age class was associated with increasing risk and a shorter time to death. Sheep released at ages ≥ 6 years had 3.4 times the hazard rate of sheep released at 1 to 3 years of age. Source population, MLH as a continuous and binary variable, year of release, and previous exposure to other pathogens were not associated with survival once we accounted for the influence of age and exposure to BRSV on time to death. Associations with sex could not be evaluated in the modeling procedure because males were more likely to be lost to follow up. Evaluation of the proportional hazards assumption for the final model based on a test of Schoenfeld residuals indicated that the relative risk for each variable of interest was the same in time for the duration of the study (P = 0.322).

**Table 2:** Factors significantly related to time to death in the final Cox proportional hazards model of survival of bighorn sheep translocated to the San Andres Mountain, New Mexico, USA.

Factor	Hazard Ratio	**Std. Err**.	Z	P>|z|	95% Conf. Interval
Age 3 to <6 yr ^a^	1.47	0.664	0.85	0.397	0.60 - 3.56
Age ≥6 yr ^a^	3.37	1.75	2.34	0.019	1.22 - 9.32
BRSV antibodies ^b^	0.37	0.18	-2.03	0.043	0.14 - 0.97

^a ^Youngest age class (1 to <3 years old) designated as reference category

^b ^BRSV = bovine respiratory syncitial virus

## Discussion

A key challenge for conservation biologists is to determine the most appropriate demographic and genetic management strategies for wildlife populations threatened by disease [17-19].

Bighorn sheep in North America provide a useful model for examining this issue because they are highly susceptible to infectious diseases, and they are frequently translocated to re-establish or augment populations. In this study, we took advantage of a management effort - the restoration of bighorn sheep to the SAM - to test whether genetic background and previous pathogen exposure influenced the survival of translocated bighorn sheep.

Variability and local adaptation are considered to be key genetic factors influencing the persistence of small populations of bighorn sheep [10,20,21], and this strongly influenced the choice of source animals for the SAM translocation. Since the RRWA herd was founded with animals from the SAM, their translocation presumably maximized the retention of locally adapted gene complexes. However, the RRWA herd had low genetic variability (mean MLH = 0.39; Table 1) because it had been managed for >20 years as an inbred population. In contrast, the KNWR population was much more diverse (mean MLH = 0.57-0.60; Table 1), ensuring that the newly established SAM population would be more variable than one established only with animals from the RRWA captive population.

While the genetic management goal of achieving increased heterozygosity in the new SAM population was accomplished, it was not associated with enhanced survivorship of the individual sheep that were translocated. Bighorn sheep from the KNWR had significantly higher genetic diversity than those from the RRWA, but neither source population nor genetic background (MLH) influenced their survival in the SAM. Our failure to detect a significant relationship between genetic diversity and adult survival is consistent with the Chapman et al. [13] meta-analysis of HFC studies. In their comprehensive analysis, Chapman et al. [13] concluded that the effects of such correlations are very weak, and that other proposed measures of genetic variation (SH, IR) are no more powerful than MLH for detecting relationships. We acknowledge that a larger sample size and more marker loci would have increased the power of our analysis, but any such effect is apparently very weak. Our failure to detect a fitness effect with neutral markers (microsatellites) is perhaps not surprising given that Gutierrez et al. [22] failed to find a strong association between MHC variation and disease resistance in bighorn sheep.

Previous examination of genetic variability among bighorn sheep suggests that microsatellite diversity has been influenced primarily by neutral factors, and MHC diversity by balancing selection [6,9,22,23]. These studies have shown that genetic distances between populations are roughly proportional to geographic distance, and that most genetic variability is apportioned within, rather than between, populations. These results are best explained by isolation by distance (reduced gene flow), and provide little genetic evidence for local adaptation. Our results are consistent with this view - the RRWA animals derived from the original SAM population did not have increased survivorship. Conversely, inbreeding within the RRWA population could have reduced fitness by increasing the accumulation and expression of deleterious recessive alleles [24,25]. We found no evidence of such an effect among translocated adult sheep (survival was not associated with source population). Although we did not detect fitness differences among translocated animals, heterosis or genetic rescue effects may occur in F1 and later generations as the two source populations interbreed [25,26]. While we were able to sample nine offspring born in the SAM for genetic and pathogen analyses (Table 1), we were unable to monitor their relative fitness and they were not included in any of our survival analyses.

Pneumonia was the most frequent cause of death, accounting for 50% (17/34) of the documented mortalities. This mirrors what has been reported for many other populations, illustrating the ongoing importance of pneumonia as a cause of morbidity and mortality across the range of bighorn sheep [4,20,27,28]. Several bacteria (*Pasteurella *and *Manheimia *spp.) and viruses (BRSV, PI3) have been implicated as primary or secondary pathogens in enzootic and epizootic pneumonia, and we associate these organisms with pneumonic disease because they can be detected in sick and dead animals. However, we know far less about causal relationships. The multifactorial etiology of pneumonia, coupled with the complex interplay of host, pathogen, and environment, has made it very difficult to identify a single, specific cause of pneumonia.

Instead of focusing on identifying pathogens after death, we looked at how previous pathogen exposure influenced the future survival of healthy bighorn sheep. The presence of antibodies was used as a marker of pathogen exposure, indicating that viral transmission and infection had occurred at some point in the past. Animals translocated into the SAM showed evidence of previous exposure to BTV (RRWA only) as well as BRSV and PI3 (both RRWA and KNWR; Table 1). All animals were healthy at the time of translocation, yet previous BRSV exposure was associated with increased survival regardless of age class. This suggests that antibodies to this virus provided some level of protection against pneumonia after animals were released into the SAM.

Age class at the time of release was significantly related to survival, and younger animals lived longer than those > 6 yrs of age (Table 2). This intuitive result illustrates the demographic advantage of selecting young animals for reintroductions. Singer et al. [20] found that translocation success was directly related to the number of animals - larger translocations resulted in populations that were more likely to persist over time. However, pneumonia epizootics appear to be driven by density-dependence, and thus disease may constrain population growth and size [5]. Lethal removal of mountain lions was conducted in the SAM throughout our study period [8], likely influencing the relative proportion of mortality caused by predation versus pneumonia. In the absence of controlled studies, we have no way of inferring whether lion control increased overall survivorship. Regardless, there were no significant relationships between mean MLH and death due to lion predation or pneumonia.

## Conclusions

We conclude that increased heterozygosity did not increase survival following translocation, nor did it reduce individual susceptibility to dying specifically from pneumonia. Instead, we found that previous pathogen exposure was more important than genetic heterozygosity as a marker for predicting survival of translocated animals. In their review of > 600 published HFC effects, Chapman et al. [13] reported a publication bias with non-significant effects being under-reported in the literature. We acknowledge that confounding factors, such as age, and small sample sizes likely limited our ability to detect significant relationships between genetic variability, disease and fitness. These limitations often plague investigators who conduct observational field studies in natural settings. We encourage others to test the theoretical advantages of increased genetic variability using a variety of genetic markers in long term field studies that also evaluate ecological factors, such as disease, that are likely to influence survival. Every wildlife translocation is an experiment, but many translocations lack an experimental design. Whenever possible, translocations should be designed and evaluated to test hypotheses that will improve our understanding of how pathogen exposure and genetic variability influence fitness.

## Authors' contributions

WMB and MEW designed and implemented the study. MCTP and CKJ conducted genetic and statistical analyses, respectively. All authors have approved the final manuscript.
